# Conventional, functional and radiomics assessment for intrahepatic cholangiocarcinoma

**DOI:** 10.1186/s13027-022-00429-z

**Published:** 2022-03-28

**Authors:** Vincenza Granata, Roberta Fusco, Andrea Belli, Valentina Borzillo, Pierpaolo Palumbo, Federico Bruno, Roberta Grassi, Alessandro Ottaiano, Guglielmo Nasti, Vincenzo Pilone, Antonella Petrillo, Francesco Izzo

**Affiliations:** 1 Division of Radiology, “Istituto Nazionale Tumori IRCCS Fondazione Pascale – IRCCS di Napoli”, 80131 Naples, Italy; 2 Medical Oncology Division, Igea SpA, Napoli, Italy; 3 Division of Hepatobiliary Surgical Oncology, “Istituto Nazionale Tumori IRCCS Fondazione Pascale – IRCCS di Napoli”, 80131 Naples, Italy; 4 Division of Radiotherapy, “Istituto Nazionale Tumori IRCCS Fondazione Pascale – IRCCS di Napoli”, 80131 Naples, Italy; 5 Department of Diagnostic Imaging, Area of Cardiovascular and Interventional Imaging, Abruzzo Health Unit 1, Milan, Italy; 6 Italian Society of Medical and Interventional Radiology (SIRM), SIRM Foundation, Milan, Italy; 7 Diagnostic Imaging Section, University of Aquila, L’Aquila, Italy; 8 Division of Radiology, “Università Degli Studi Della Campania Luigi Vanvitelli”, Naples, Italy; 9 Division of Abdominal Oncology, “Istituto Nazionale Tumori IRCCS Fondazione Pascale – IRCCS di Napoli”, Naples, Italy; 10 Department of Medicine, Surgery and Dentistry, University of Salerno, Salerno, Italy

**Keywords:** ICC, Ultrasound, Computed tomography, Magnetic resonance imaging, Radiomics

## Abstract

**Background:**

This paper offers an assessment of diagnostic tools in the evaluation of Intrahepatic Cholangiocarcinoma (ICC).

**Methods:**

Several electronic datasets were analysed to search papers on morphological and functional evaluation in ICC patients. Papers published in English language has been scheduled from January 2010 to December 2021.

**Results:**

We found that 88 clinical studies satisfied our research criteria. Several functional parameters and morphological elements allow a truthful ICC diagnosis. The contrast medium evaluation, during the different phases of contrast studies, support the recognition of several distinctive features of ICC. The imaging tool to employed and the type of contrast medium in magnetic resonance imaging, extracellular or hepatobiliary, should change considering patient, departement, and regional features. Also, Radiomics is an emerging area in the evaluation of ICCs.

Post treatment studies are required to evaluate the efficacy and the safety of therapies so as the patient surveillance.

**Conclusions:**

Several morphological and functional data obtained during Imaging studies allow a truthful ICC diagnosis.

## Introduction

Primary liver cancer, including hepatocellular carcinoma (HCC), intrahepatic cholangiocarcinoma (ICC) and other rare types, is the sixth most usually detected tumour and the third leading cause of tumour death worldwide [1–3].

In line with the Liver Cancer Study Group of Japan, based on the macroscopic growth pattern, ICC can be categorised as mass forming, periductal infiltrating, or intraductal growing type [4–9]. The mass forming is the most frequent, accounting for 78% of all cases of ICC [10–12]. Periductal infiltrating tumours develop alongside the bile ducts, producing bile duct wall thickening, luminal stenosis and proximal biliary dilatation [7–9]. This type represents about 16% of all ICCs, but it is the most common at hilar side [12]. Intraductal growing CC is the rarest form [12].

ICC could have an abundant stromal fibrosis, features that is infrequent in perihilar type, but usually found in peripheral type [12]. This datum is revealed into imaging. An appropriate diagnosis is crucial since radical surgery is the only curative treatment [10]. Multiphasic contrast-enhanced computed tomography (CT) and magnetic resonance imaging (MRI) are imaging tools to choosing in ICC patients. In the pre-surgical assessment phase, a multimodal strategy should be favorite in order to use the pros and cons of CT and MRI. In fact, the high spatial resolution provided by CT with the high soft tissue contrast by MRI favours a more accurate assessment. Additionally, thanks to the ability of correlate morphological and functional features, MRI allows a suitable characterization of liver lesion. Also, the different contrast agent for MRI could influence diagnosis considering the differences between hepatobiliary agents (HBAs) and extracellular agents (ECAs) [13–21]. Also, Diffusion Weighted Imaging (DWI) has been employed to assess focal liver lesions (FLL). [14].

When the patient is unfit for surgical resection, locoregional therapies should be considerated in order to delay disease progression and prolong life or as a bridge to curative treatment (i.e. downstaging). For ICCA, trans-arterial chemoembolization (TACE), drug-eluting bead trans-arterial chemoembolization (DEB-TACE), radiofrequency ablation (RFA), microwave ablation (MWA), trans arterial-radioembolization (Yttrium90; TARE), and reversible electroporation have been assessed [21–26]. Several researches have shown that these treatments, alone or in combination with chemotherapy, improve Overall Survival (OS) [24]. In a cohort of non surgical treated small (≤ 5 cm) iCCA, staged as AJCC I/II, RFA-treated patients showed better survival respect to chemoradiotherapy [23], results reported by a recent meta-analysis, too [24]. Consequently, RFA is the treatment of choice in small ICCs. In this scenario, assessment post treatment is a new challenge for radiologists [25–29].

In this narrative review we reported an overview and update on ICC assessment in staging and post treatment phase.

## Methods

This study is autonomous without protocol and registration number.

We analysed several electronic datasets: PubMed (US National Library of Medicine, http://www.ncbi.nlm.nih. gov/pubmed), Scopus (Elsevier, http://www.scopus.com/), Web of Science (Thomson Reuters, "http://apps.webofknowledge.com) and Google Scholar (https://scholar.google.it/), using the subsequent keywords: “ICC” AND “Ultrasound”, “ICC” AND “Computed Tomography”, “ICC” AND “Magnetic Resonance Imaging”, “ICC” AND “Radiomics”, “ICC” AND “Li-RADS”, “ICC” AND “Ablative Therapies” AND “Assessment”.

Papers published in English language has been scheduled from January 2010 to December 2021. All titles and abstract were assessed, and full text was retrieved for each included article. Clinical studies (eg. retrospective analysis, case series, prospective cohort study) on morphological and functional diagnostic assessment in staging and post treatment phase on ICC were retrieved. Exclusion criteria were inaccessibility of full text, case report, review, or letter to editors.

## Results

We recognised 2201 pertinent papers. After deleting 1588 duplicates, we acquired 613 papers. We recognised 28 papers throughout visualizing reference lists of the aquired papers that we added to the 633 papers selected (total number of scrutinized articles was 641). Then we rejected 553 inapppropiate papers throughout reading abstracts.

**Fig. 1:**
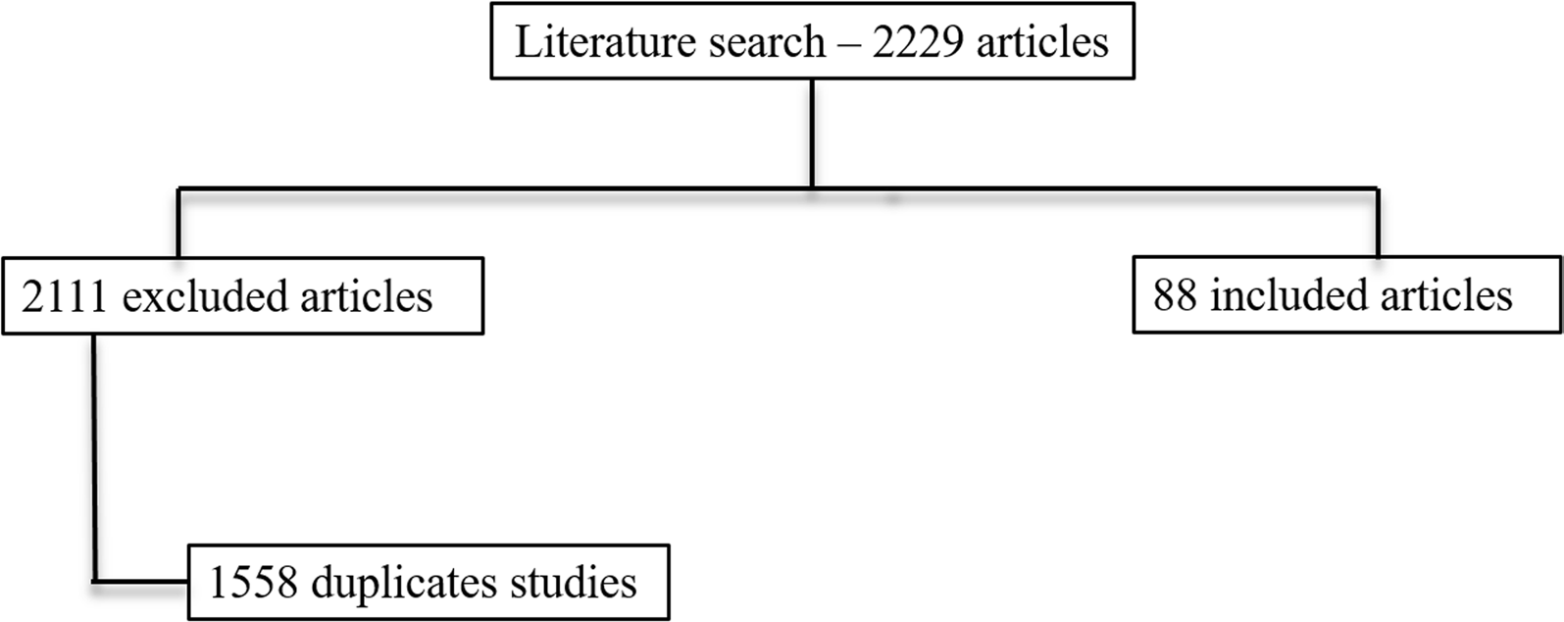
Included and excluded studies in systematic review

A total of 88 clinical studies were assessed in these narrative review. The reference flow is summarized in the study flow diagram (Fig. 1).

## Discussion

### Diagnostic tools

#### Ultrasound

Ultrasound (US) is an imaging tool inexpensive, non-aggressive, non based on X-rays suorces and so repeatable [30–40]. The possibility of injecting a contrast medium (Contrast-Enhanced Ultrasound (CEUS)) determines a technical evolution [30]. CEUS has a wide range of applications, being able to emphase the large vessel flows and the microcirculation; therefore it has as its particular target the oncological setting [30–40]. The liver contrast study during CEUS includes: (1) arterial phase, which starts 10–20 s and ends 30–45 s after contrast injection; (b) portal venous phase, which lasts from 30–45 s to 2 min after contrast agent injection; and (c) the late phase, which lasts from 4 to 6 min after the contrast injection. It is possible to assess a post-vascular phase if used Sonazoid; during this phase is possible to assess the uptake of the contrast medium by Kupffer cells, in order to obtain functional parameters.

US with CEUS allows the first assessment of FLLs, helping in the differentiation between benign lesions (as haemangiomas) and malignant tumours.

**Fig. 2:**
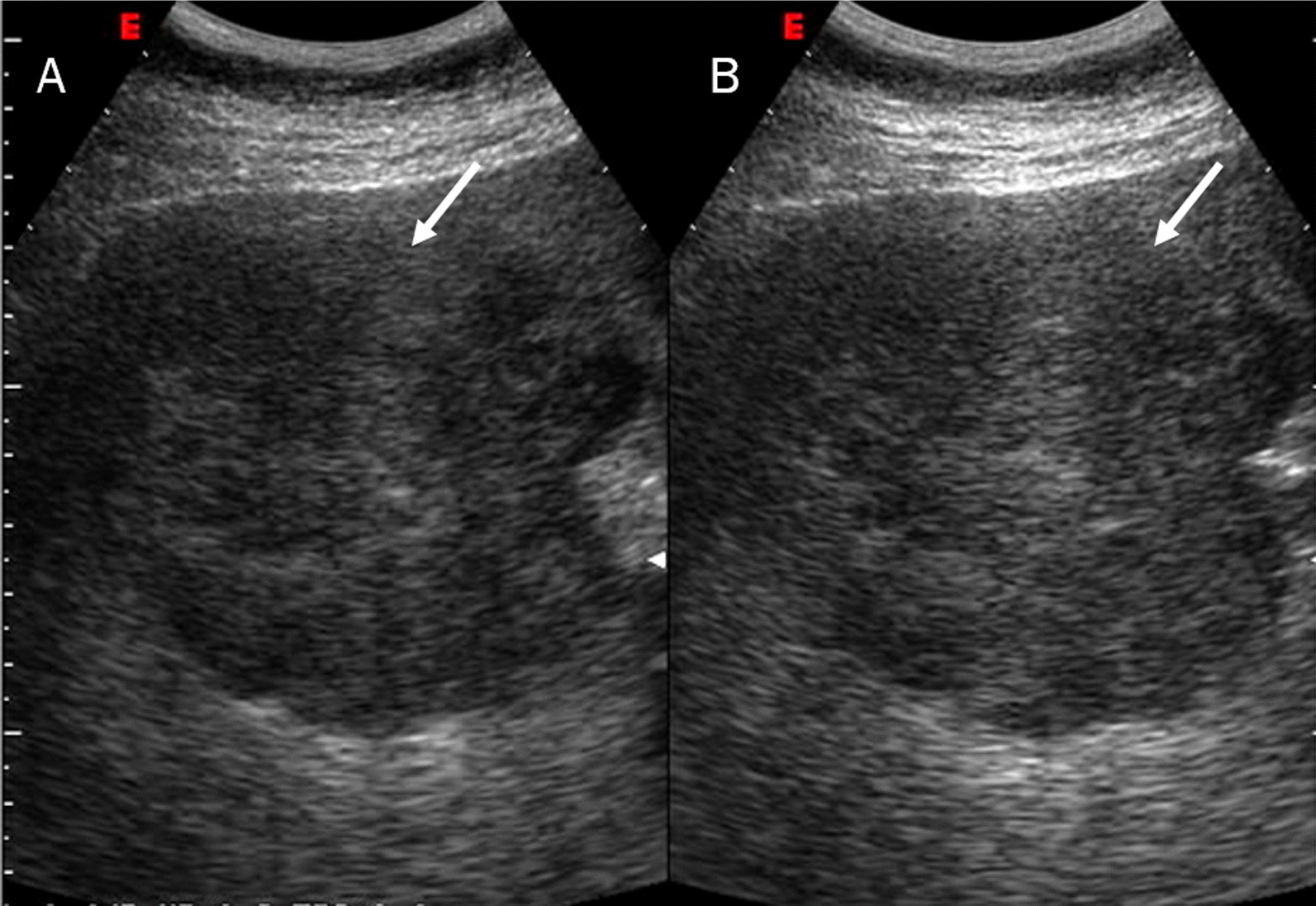
US assessment of ICC on VIII-VII hepatic segment. The lesion (arrow) is sizeable mass of lobulated contour with heterogeneous echogenicity due to the interleaving of necrosis, fibrosis and active growth tissue

**Fig. 3:**
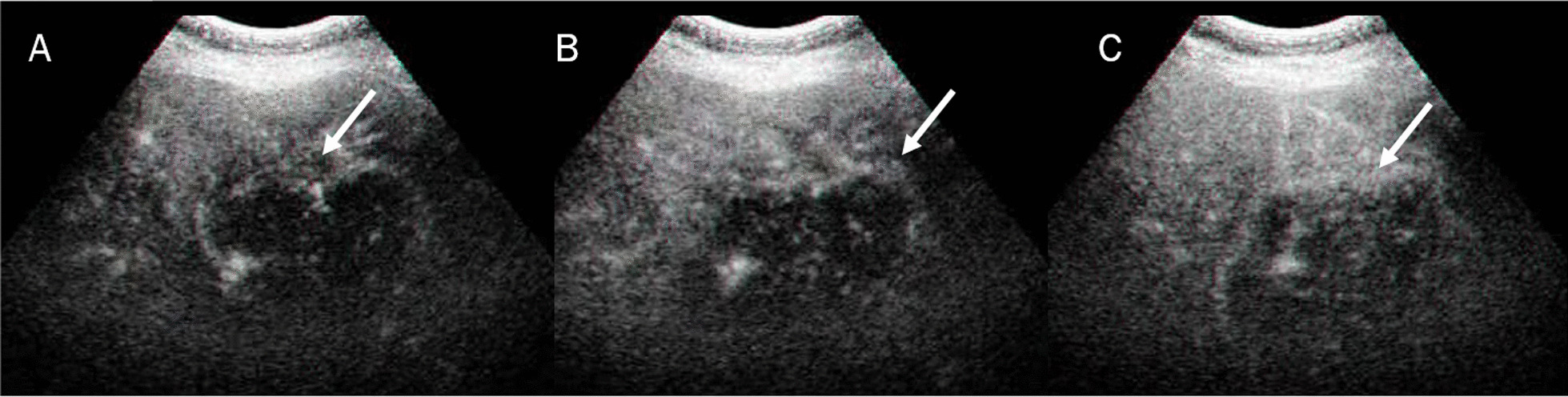
CEUS assessment of ICC on V hepatic segment. The lesion shows peripheral rim hyperenhancement in arterial phase (**A**) and eraly (**B**) and marked (**C**) washout

Mass-forming ICC, on US studies, occurs as a large non-encapsulated mass with lobulated or variable shape. As associated features, it is possible see hepatic capsular retraction and dilated peripheral bile ducts (Fig. 2), so as satellite lesions, near the primary lesion, and liver metastases. Necrosis, fibrosis and active growth tumour can cause lesion heterogeneous echogenicity [40]. During contrast study, ICC could show hyperenhancement during arterial phase (Fig. 3), with washout in late phase [40]. There is little agreement regarding the differential diagnosis between ICC and HCCC, on CEUS. ICC exhibits lesser enhancement in arterial phase with early (< 60 s) and prominent washout compared to HCC [40–54]. Chen et al. demonstrated that the pooled sensitivity of CEUS in differentiating ICC from HCC was 0.92 and the pooled specificity was 0.87 [42].

The advantage of US/CEUS is the wide diffusion, the possibility to guide ablative procedures and to assess immediately the necrosis. In this scenario, the radiologist should be confident with this tool.

#### Computed tomography

Computed tomography is habitually the first diagnostic toll in the assessment of FLLs [55–63]. The post contrast enhancement patterns of FLLs are critical for categorizing lesions, and the CT image analysis, during characterization phase, allows the diagnosis of FLLs [55]. Yet, in modern clinical setting, this analysis could be prejudicate by the radiologist’s skill [61, 64–69].

CT is frequently the first diagnostic imaging technique to characterize ICC and contrarily to US/CEUS evaluation it is operator independent [70–79]. Although, Chen et al. [71] compared the enhancement pattern of ICC on CEUS with that on CT in 40 pathologically proven ICC lesions. The enhancement level and pattern in the post contrast phases on both CEUS and CT were assessed. CEUS made a correct diagnosis in 32 lesions while CT in 27 lesions. So, they demonstrated that CEUS had the same accuracy as CT in ICC diagnosing [71]. However, CT not only offers a complete evaluation of tumour, but also the association between the lesion and neighbouring structures as the hepatic artery, portal vein and biliary tree, so as the whole body surveillance for metastases assessment [53]. According to a recent meta-analysis, CT provides a satisfactory pooled sensitivity of 89% and pooled specificity of 92% for portal vein involvement and 84% pooled sensitivity and 93% pooled specificity for hepatic artery involvement in perihilar CC [77].

**Fig. 4:**
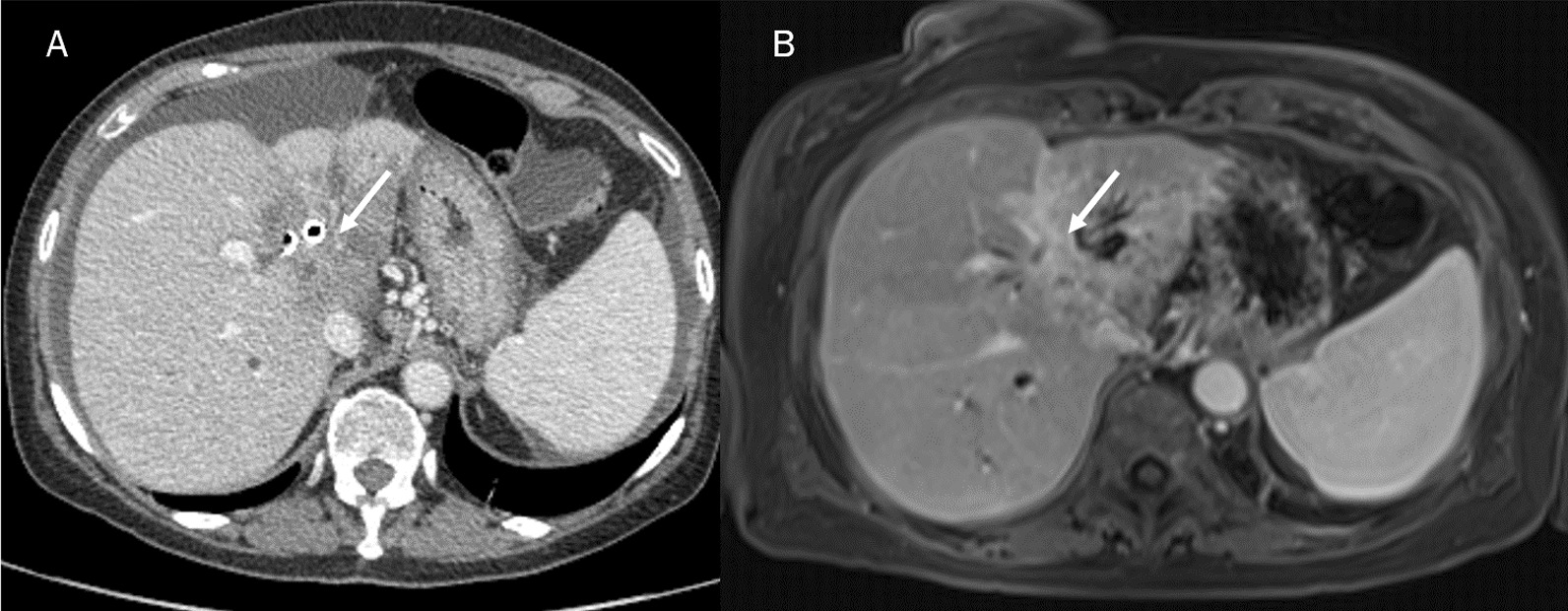
CT (**A**) and MRI (**B**) portal phase assessment of ICC. The typical contrast enhancement is a gradual centripetal enhancement on dynamic studies (arrows)

Typical study protocol includes several phases with and without contrast injection: non-contrast phase, arterial, portal or venous, and delayed phase, which is obtained from 3–5 min to 30 min after contrast medium injection [53]. The non contrast phase allows the detection of intraductal stones. Arterial phase, performed 20–30 s after contrast medium injection, allows to assess arterial anatomy and to plan the surgical approach. Venous phase is performed 25–30 s after the completion of late arterial phase scanning. The usual CT pattern of a mass-forming ICC is a hypo-attenuated mass with irregular peripheral enhancement in the hepatic arterial phase, and gradual centripetal enhancement during venous and delayed phases (Fig. 4) [53]. This enhancement pattern is due to the histologically characteristic of the lesion, with the peripheral tissue which comprises viable cells, whereas the central portion comprises coagulative necrosis and fibrous stroma [53].

Latest techniques have been established to rise tumour detection, such as dual-energy CT (DECT) [80–92]. DECT, which is founded on instantaneously acquisition of two image datasets at different energy levels, can produce virtual monochromatic images (VMIs) [80]. Several reseaches demonstrated that DECT allowed considerably better focal liver lesion detection compared to conventional CT [93]. Additionally, radiation and contrast media dose decrease would mainly be beneficial for patient surveillance. Infact, radiation exposure is often underestimated in oncologic patients due to their relatively short life expectancy and the clinicians' focus on the immense benefit of early detection of recurrence [60–63].

Several researches assessed the role of DECT in the characterization of ICCS, with regard to the use of DECT quantitative parameters compared with the use of conventional CT for differentiating small (≤ 3 cm) intrahepatic mass-forming cholangiocarcinoma (IMCC) from small liver abscess (LA) during the portal venous phase (PVP) which showed greater accuracy than quantitative and qualitative analyses of conventional CT in these lesions [94, 95].

Perfusion CT (pCT) is a diagnostic method founded on contrast kinetics of tissue that provides quantitative data. pCT assesses dynamic variations in tissue iodine concentration over time, allowing to obtain tissue-specific data, including blood flow (BF), blood volume (BV), time to peak concentration (TTP), vascular permeability surface area product (PS), and permeability (*K*trans), which can be utilised as biomarkers for tumour vascularization and perfusion. Several authours showed that pCT is a considerable tool for lesion characteritation, treatment response assessment, and prognosis [96–101].

Zhao et al. [98] evaluated different perfusion parameters and corresponding histogram parameters compared to conventional post contrast CT analysis in differentiating ICC from HCC. They demonstrated that the mean value, and all the percentiles of the arterial enhancement fraction (AEF) were significantly higher in HCC than in ICC. The variance in hepatic arterial blood supply perfusion (HAP) and AEF for the mean perfusion data and all percentile parameters between lesion and normal liver were drastically higher in HCC than in ICC. The relative AEF was statistically significant between HCC and ICC. Among all parameters, the mean value of ∆AEF, the 75th percentiles of ∆AEF and rAEF, and the 25th percentile of HF_tumor_ had the highest sensitivities of 94.4%, while the 50th percentile of rAEF had the highest specificity of 82.4%. AEF (including ΔAEF and rAEF) and the corresponding histogram parameters derived from triphasic CT aided accurate lesion diagnosis [98].

The CT is the most widley used tool in the assessment of FLLs, and the possibility to utilised new techniques, as DECT or perfusion, allow to choose the more appropriate study protocol considering patients and conventional features of lesions.

#### Magnetic resonance imaging

MRI is the more suitable tool in the assessment of FLL, thanks to its possibility to analyse functional and morphological data [102–115].

#### Morphological assessment

**Fig. 5:**
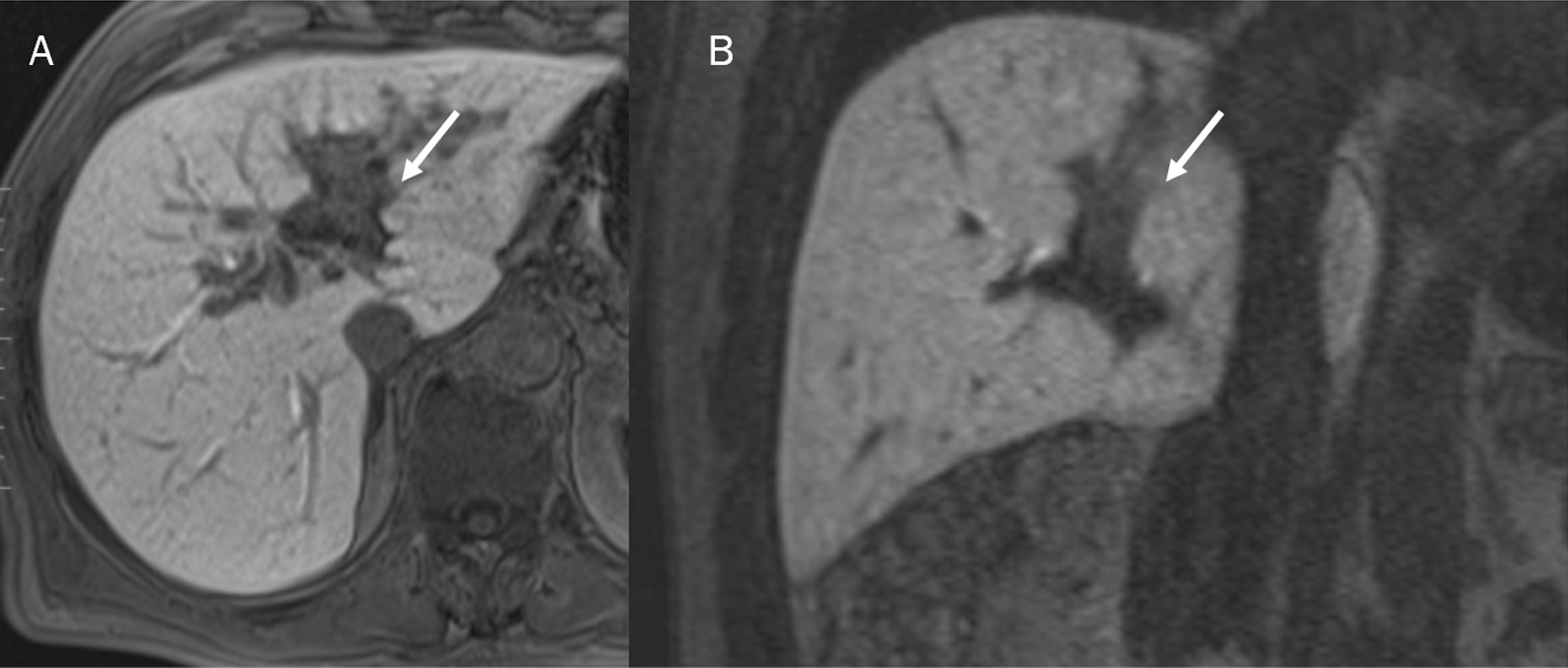
MRI assessment of ICC in hepatobiliary phase of contrast study. The lesion (arrows) shows hypointense signal intensity in **A** (axial plane) and **B** (coronal plane)

Several morphological pattern allow to identify ICCs. Granata et al. assessed MR features of 88 ICCs (61 mass-forming type; 23 periductal infiltrating tumours type and 4 intraductal growing type), showing that on T1-W sequences, among the 61 with mass-forming ICCs, 48 (78.7%) had targetoid appearance and 13 (21.3%) hypointense signal. On T2-W sequences, 48 lesions had targetoid appearance while 13 hyperintense signal. After the injection of contrast medium, all lesions showed Rim Arterial Hyperenhancement (APHE), during arterial phase and progressive contrast enhancement with non-peripheral washout appearance in portal phase. In transitional phase and hepatobiliary (EOB) phase 48 mass-forming ICCs showed targetoid appearance and 13 (21.3%) showed hypointense SI (Fig. 5) [12]. They demonstrated that mass-forming ICCs are more likely to exhibit targetoid appearance in T1-W, T2-W and DWI sequences, Rim APHE during arterial phase, progressive contrast enhancement and non-wash-out appearance in portal phase with targetoid appearance (TA) in transitional and hepatobiliary Phase (HB) phase, allowing the differentiation between study group and each other control group [12].

The TA is correlated to central fibrous stroma, which appears as an area of lesser intensity, compared to a more hyperintense peripheral area on T2-W sequences. The presence of this central fibrous stroma also typifies the vascular patterns during the post contrast studies [60, 116].

#### Dynamic contrast-enhanced (DCE)-MRI

DCE-MRI offers functional informations on perfusion, vessel permeability and extracellular-extravascular space composition by assessing the differences in Signal Intensity (SI) during dynamic contrast studies [117–122]*.* DCE-MRI can be analysed qualitatively, semi quantitatively and quantitatively. Quantitative analysis comprises the assessment of the pharmacokinetics of an administered contrast medium [117–122]. The largely assessed parameter is the volume transfer constant, Ktrans. The quantitative method shows several weakness as high output flexibility, poor consistency since correlated to several variables and different models used [122]. Qualitative approach (qMRI) is based on time–intensity curve (TIC) visual analysis. The main weakness of this approach is the ROI placing [122–130]. Semi-quantitative method evaluates TIC shape parameters offering data on tumour biology [122].

Considering that the liver is a dual-blood-supply organ and that this can affect the quantitative DCE-MRI obtained parameters, however, in ICC patients, it has been demonstrated that pertinet DCE-MRI data can assess angiogenesis, helping diagnosis treatment response evaluation. Tan et al. assessed 50 ICC patients, analysing the rate constant (Kep), extravascular space volume fraction (Ve), and tissue volume transfer constant (Ktrans). There showed that Ktrans, Kep, and Ve values were all not correlated with pathological classification [126]. Zhou et al. demonstrated that radiomics signature derived from DCE-MRI can be a consistent biomarker for callculating MVI of ICC [127]. Lin et al. found that kinetic model is a new and feasible method to differentiate HCC from ICC in pre-transplantation setting [128]. Konstantinidis et al. showed that in ICC patients subjected to hepatic arterial chemotherapy infusion, tumour perfusion DCE.MRI parameters were higher in ≥ 3-year survivors and permitted to distinguish this group from < 3-year survivors [130].

#### Diffusion weighted imaging-MRI

DWI provides functional quantitative parameters on the tissue’s microstructure valuating water proton mobility. Water diffusion mobility is linked to cell density, vascularity and viscosity of tissues and it’s possible to obtain biomarkers employing a mono-exponential or bi-exponential approach [130–142]. Intravoxel Incoherent Motion method (IVIM) is a bi-exponential approach, which allow to obtain the pure tissue coefficient (Dt) linked only to diffusion water mobility, the pseudo-diffusion coefficient (Dp) linked to blood mobility, and the perfusion fraction (fp) [143–148]. The convential DWI method is based on the theory that water diffusion follows a Gaussian motion. However, water molecule diffusion within biologic tissue exhibits non-Gaussian behavior. Jensen et al. in 2005 suggested a non-Gaussian diffusion model named Diffusion Kurtosis imaging (DKI) [149]. Using DKI it is possible to obtain kurtosis median coefficient (MK), which measures the tissue diffusion deviation from a Gaussian model, and the mean value of the diffusion coefficient (MD) with the correction of the non-Gaussian bias [149–151].

**Fig. 6:**
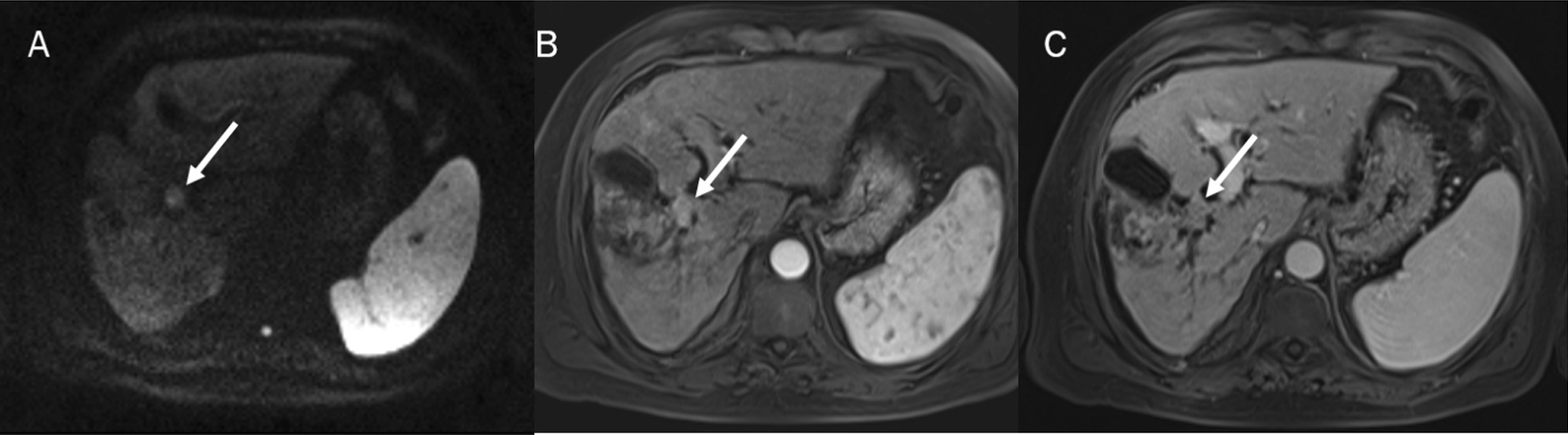
In DWI (**A**: b 800 s/mm2)) the lesion shows restricted signale and arterial hyperenhancement (**B**) with progressive enhancement (**C**) during contrast stuy

**Fig. 7:**
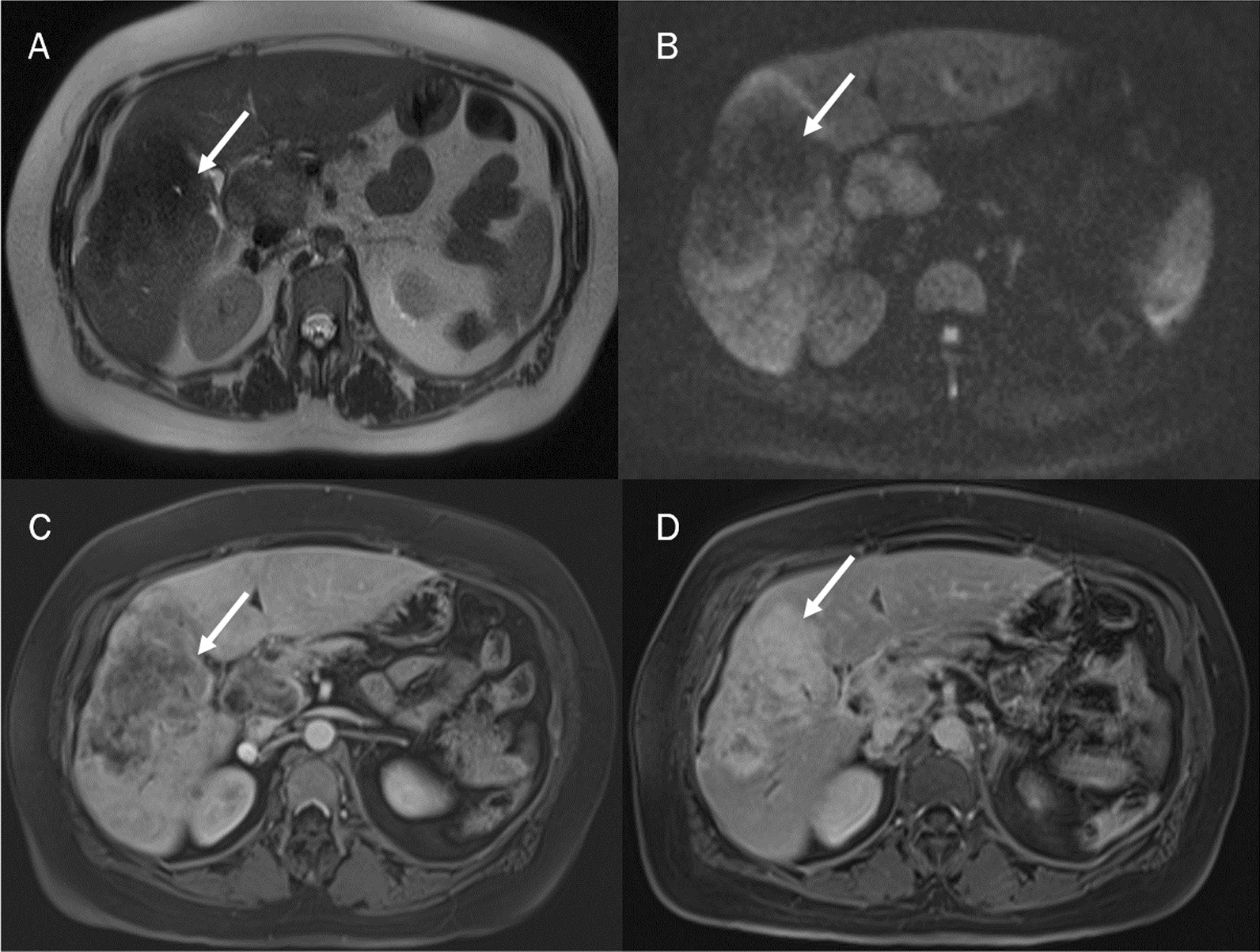
The targetoid appearance (TA) due to the fibrous stroma in the center of the ICC appears as a central area of less intensity, compared to a more hyperintense peripheral area on T2-W sequences (**A**) and DWI (**B**). The presence of this central fibrous stroma also characterizes the behaviour during the dynamic study. On dynamic contrast study, the mass-forming ICCS showed prominent peripheral rim enhancement (**C**: arterial phase) with centripetal or gradual progressive enhancement (**D**: delayed phase)

**Fig. 8:**
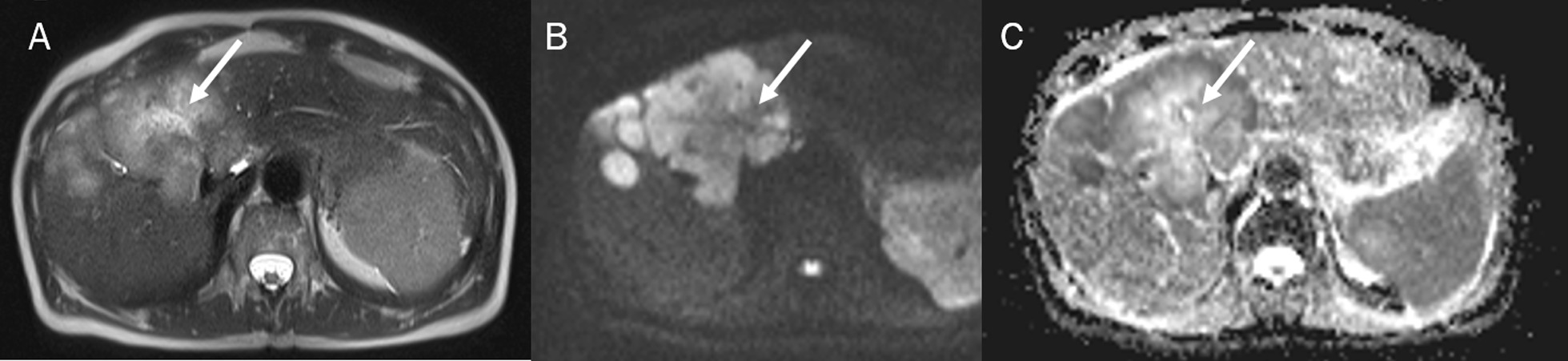
MRI assessment of liver colon rectal metastases. The lesions show (arrow) the TA appearance due to the central necrosis, with a central area of higher intensity compared to peripheral area of lesion on T2-W sequences (**A**) and DWI sequences (**B**, **C**)

The role of DWI in ICC has been evaluated in different studies (Fig. 6). Xu et al. showed that DWI detection rates were higher than those of the morphological sequences alone and there were significant differences between the Apparent Diffusion Coefficient (ADC) values of the lesion edge, lesion centre and liver parenchyma [152]. Kovač et al. demonstrated that univariate analysis shown that several findings suggested ICC diagnosis over metastases: lobulated shape, heterogeneous T2W signal intensity, capsular retraction, segmental biliary dilatation, target sign on DWI and rim-like enhancement on arterial phase followed by progressive enhancement in delayed phases. ADC values measured in the periphery of the lesion were significantly lower in ICC. Multivariate analysis showed that TA on DWI was the most significant predictor of ICC (Fig. 7 and 8) [153]. This result was confirmed by several researches and in differential diagnosis between ICC and HCC, TA was the parameter which allowed it [154–159]. Kovač et al. [155] assessed IVIM-derived parameters, showing that D could help in differentiation between ICC and metastasis. Shao et al. demonstrated that ADC, *D*, and *f* values could help ICC from HCC [160]. These results were obtained also by Peng et al., which showed that ADC and D_slow_ were significantly lower in the HCC group than in the ICC group, while D_fast_ was significantly higher in the HCC group than in the ICC group; f did not significantly differ between the HCC and ICC groups [161].

At the best of our knowledge no data on non-Gaussian diffusion behaviour has been described in ICC patients.

#### BOLD-MRI

Hypoxia is developing key factor of the aggressive tumour biology and the relative resistance to conventional as well as targeted therapies [162]. The only non-invasive diagnostic tool that could reproduce in clinical setting blood oxygen level is blood oxygen level- dependent (BOLD) functional MRI (fMRI). BOLD MRI makes use of paramagnetic properties of deoxyhemoglobin and is mainly used for regional quantification of oxygenation [162, 163].

To date, only Park et al. assessed BOLD in ICC. They evaluated 100 patients (43 HCCs, 36 metastases, 17 ICCs, and 23 haemangiomas). BOLD MRI was performed using a multiple fast-field echo sequence to generate 20 T2*-W images. The T2* value of each tumour were calculated. They demonstrated that the mean T2* value (ms) of haemangiomas (97.3 ± 20.2) was the highest, followed by HCCs (48.4 ± 12.7), metastases (37.1 ± 10.5), and cholangiocarcinoma’s (36.6 ± 11.1). These values were significantly different (haemangioma vs. others tumours and HCC vs. metastasis or cholangiocarcinoma) (*p* ≤ 0.001). The agreement between the T2* colour map and dynamic images was moderate for all tumours (k = 0.544), good for tumours > 2.0 cm (k = 0.666), and fair for tumours ≤ 2.0 cm (k = 0.334). With the gadoxetic acid-enhanced MRI used as a reference, the sensitivities of BOLD MRI (T2* colour map) for displaying hypervascularity of HCC (categories of 1–3) were 81.0% (n = 34/42) and 78.6% (n = 33/42) for both observers [163].

Today, MRI is unique compared to CT and US, since it allows during only one protocol study to assess conventional data, obteinde by T2-W and T1-W sequences with functional data, obtained by DWI, DCE-MRI and Bold sequences. In this scenario MRI not only is a problem solving, but the first toll that should be used in patients with FLLs.

#### Radiomics analysis

Radiomics is a rapidly evolving field of research concerned with the extraction of quantitative data, the so-called radiomic features, within medical images. Radiomic features capture tissue and lesion characteristics such as heterogeneity and shape and may, alone or in combination with demographic, histologic, genomic, or proteomic data, be used for clinical problem solving [73, 164–175]. Radiomics could hypothetically assist cancer detection, prognosis evaluation, response to treatment assessment, monitoring of disease status [176–179]. Radiomic is planned to be utilysed in precision medicine decision support, employng standard of care images that are usually obtained in clinical setting [73, 166–170]. Moreover, Radiomics offers prognostic biomarker which allow for a fast, low-cost, and repeatable means for longitudinal analysis [171–179].

To date, in several studies have been assessed the role of radiomics or radiogenomics in ICC [72, 79, 180–204]. The area of main interest has been the evaluation of recurrence after surgical resection [72, 79, 180–187]. Chu et al. [79] evaluated 203 ICCs, that were allocated with a ratio of 7:3 into the training cohort and the validation cohort. Clinical characteristics and radiomics features were selected using random forest algorithm and logistic models to construct a clinical model and a radiomics model, respectively. The radiomics model showed a higher AUC than the clinical model in the validation cohort for predicting futile resection in ICC. The radiomics model reached a sensitivity of 0.846 and a specificity of 0.771 in the validation cohort. Moreover, the radiomics model had comparable AUCs with the combined model in training and validation cohorts [79]. Quin et al. [180] developed a multilevel model, integrating clinicopathology, molecular pathology and radiology to predict early recurrence after curative surgery, using a machine-learning analysis of 18,120 radiomic features based on CT constrast studies and 48 clinico-radiologic features. They showed that the radiomics-based multilevel model had superior performance to rival models and conventional staging systems, and could serve as a visual prognostic tool to plan surveillance and guide post-operative individualized management [180]. Also Hao et al. [181] developed a non-invasive CT based radiomics analysis model to predict early recurrence in 177 ICC patients. Radiomic features were extracted on CT images. Six established radiomics models were selected as stable ones by robustness-based rule. Among those models, Max-Relevance Min-Redundancy (MRMR) combined with Gradient Boosting Machine (GBM) yielded the highest AUCs of 0.802 and 0.781 in the training and test cohorts, respectively [181]. Xiang et al. [72] developed a radiomics prediction model based on contrast-enhanced CT to distinguish Microvascular invasion (MVI) in ICCs. Also, Zhou et al. [127] develop a radiomics signature for preoperative prediction of MVI in ICC patients. They evaluated the data obtained based on DCE-MR images.

An area of current interest is the possibility of identifying ideal patients for ablative treatment. Mosconi et al. [182] assessed the relationships between CT textural features prior to TARE and objective response (OR), progression-free survival (PFS), and overall survival (OS). Of the 55 patients, 53 had post-TARE imaging available, showing OR in 56.6% of cases. Texture analysis showed that iCCs showing OR after TARE had a higher uptake of iodine contrast in the arterial phase (higher mean histogram values, *p* < 0.001) and more homogeneous distribution (lower kurtosis, *p* = 0.043; GLCM contrast, *p* = 0.004; GLCM dissimilarity, *p* = 0.005, and higher GLCM homogeneity, *p* = 0.005; and GLCM correlation *p* = 0.030) at the pre-TARE CT scan. A favourable radiomic signature was calculated and observed in 15 of the 55 patients. The median PFS of these 15 patients was 12.1 months and that of the remaining 40 patients was 5.1 months (*p* = 0.008) [182].

Nodal status has also been investigated [196, 197].

The merit of this new tool is that it is able to get digital data from medical imaging and when performed under appropriate protocols, is more robust and reproducible. Nevertheless, there are remaining issues for clinical setting. First, reproducibility is a very important issue. This is correlated to several features, as acquisition protocol, method of segmentation, method for extracting imaging features, and acquisition of clinical and genomic data [102, 198–205].

#### ICC and Li-RADS: LR-M

The American College of Radiology (ACR) estabilished the Liver Imaging Reporting and Data System (LI-RADS) in patients at risks for HCC in 2011, which has been advanced over multiple updates to version 2018. In 2013, it has been introduced the concept of LR-OM for non-HCC malignancies, which was renamed as LR-M in 2014 [206–217]. Also, ACR introduced the CEUS LI-RADS in 2016 and modified LR-M observations in 2017 [211, 212]. Several researches demonstrated the role of LR-M observations for differentiating non-HCC malignancies from HCCs. An et al. [214] showed that CT and MRI observations had similar accuracy for discriminating non-HCC malignancies from HCC, with pooled accuracies of 79.9 and 82.4% for categorizing LR-M. Kim et al. [215] showed that non-HCC malignancies could be differentiated from HCCsw ith a sensitivity of 89% and a specificity of 48% by employing LR-M criteria of v2018 at gadoxetate-enhanced MRI. Zheng et al. [216] assessed that CEUS LI-RADS showed a sensitivity of 89% and a specificity of 88% for LR-M category.

Multiphase contrast studies are required to evaluate LI-RADS features. For therapy-naive lesions submitting to CT, non contrast phase is optional, while it is necessary in the post treatment setting. Late arterial phase is strongly preferred over early arterial phase [217].

CEUS is appropriate as problem solving, classifyng observations, and distinguishing tumour in vein from bland thrombus [217–220].

#### Post-treatment assessment

The primary endpoint of locoregional therapies is to obtain a complete necrosis of liver tumour that is correlated to produce a safety margin of at least 10 mm round the external margin of the tumour. RFA is a hyperthermic method causing necrosis thanks to thermocoagulation. Using RFA, the area of active tissue heating is limited to few millimetres near to electrode, with the residue of the target being heated via thermal conduction [22].

Post-ablation imaging is necessary to assess the efficacy and the safety of the treatment [22], and should be done at programmed times to evaluate response and to detect new lesions so as possible complications. Though there is no usually recognised post-treatment surveillance procedure, it should comprise 1-, 3-, 6-, 9-, and 12-month CT or MRI follow up, as for HCCs [3]. Additionally, post treatment features should be problematic to comprehend considering target location, employed treatment, used response criteria. In this scenario, imaging observations are correlated to the type and the method of therapy delivery, the timing of treatment, and the imaging technique being used to observe the effects [28].

## Conclusions

Several morphological and functional data obtained during imaging studies allow a truthful ICC diagnosis. The choice of modality (CT, US/CEUS or MRI) and MRI contrast agent (extracellular or hepatobiliary) is correlated to patient, departement, and regional features. MRI allows to correlate morphological and functional data in the ICC assessment. Also, Radiomics is an emerging field in the assessment of ICC patients.

Post treatment imaging is necessary to evaluate the efficacy and the safety of therapies so as patient surveillance. Imaging observations are correlated to the type and the method of therapy delivery, the timing of treatment, and the imaging technique being used to observe the effects.

## Data Availability

Not applicable.
