# Correction: Conventional, functional and radiomics assessment for intrahepatic cholangiocarcinoma

**DOI:** 10.1186/s13027-022-00438-y

**Published:** 2022-05-11

**Authors:** Vincenza Granata, Roberta Fusco, Andrea Belli, Valentina Borzillo, Pierpaolo Palumbo, Federico Bruno, Roberta Grassi, Alessandro Ottaiano, Guglielmo Nasti, Vincenzo Pilone, Antonella Petrillo, Francesco Izzo

**Affiliations:** 1 Division of Radiology, “Istituto Nazionale Tumori IRCCS Fondazione Pascale – IRCCS di Napoli”, 80131 Naples, Italy; 2 Medical Oncology Division, Igea SpA, Naples, Italy; 3 Division of Hepatobiliary Surgical Oncology, “Istituto Nazionale Tumori IRCCS Fondazione Pascale – IRCCS di Napoli”, 80131 Naples, Italy; 4 Division of Radiotherapy, “Istituto Nazionale Tumori IRCCS Fondazione Pascale – IRCCS di Napoli”, 80131 Naples, Italy; 5 Department of Diagnostic Imaging, Area of Cardiovascular and Interventional Imaging, Abruzzo Health Unit 1, Milan, Italy; 6 Italian Society of Medical and Interventional Radiology (SIRM), SIRM Foundation, Milan, Italy; 7 Diagnostic Imaging Section, University of Aquila, L’Aquila, Italy; 8 Division of Radiology, “Università Degli Studi Della Campania Luigi Vanvitelli”, Naples, Italy; 9 Division of Abdominal Oncology, “Istituto Nazionale Tumori IRCCS Fondazione Pascale – IRCCS di Napoli”, Naples, Italy; 10 Department of Medicine, Surgery and Dentistry, University of Salerno, Salerno, Italy

## Correction to: Infectious Agents and Cancer (2022) 17:13. 10.1186/s13027-022-00429-z

Following publication of the original article [1], the authors reported an error in the ‘Availability of data and materials’ section.

The statement in the ‘Availability of data and materials’ section originally read: Not applicable.

The statement in the ‘Availability of data and materials’ section should read: The data are available at https://zenodo.org/record/6368235#.YkKjNShBy3A.

The original article [1] has been updated.
